# Microscopy: looking into the mirror

**DOI:** 10.1038/s41377-018-0010-4

**Published:** 2018-05-18

**Authors:** Xusan Yang, Peng Xi

**Affiliations:** Department of Biomedical Engineering, College of Engineering, Peking University, Beijing, China

**Mirrors can create a virtual excitation source for optical microscopy, which can greatly enhance the spatiotemporal resolution of different fluorescence microscopy techniques, thus advancing toward long-term live cell imaging.**

In recent decades, many new discoveries have been obtained using novel optical microscopic techniques, such as confocal, multiphoton, super resolution, and light sheet microscopies, which have attracted intensive interest from biologists working in various fields. However, advances in live cell fluorescence microscopy are facing multiple challenges, such as low resolution, poor signal-to-background ratio (SBR), insufficient imaging speed, and phototoxicity. Interestingly, these grand challenges share a common solution: mirrors. When placing a reflective mirror after an objective, the beam can be reflected, and a “virtual” excitation source can be generated without additional cost. This conceptually simple approach provides an easy solution to the abovementioned challenges, such as greater signal, better contrast, improved optical section ability at relative low cost, and facilitating live cell imaging with improved spatial resolution at a high speed.

Recently, an article published by Hari Shroff’s group from National Institute of Biomedical Imaging and Bioengineering reported an approach, in which mirror reflective imaging improved the resolution, speed, and collection efficiency in dual-view light sheet fluorescence microscopy (dual-view LSFM), as shown in Fig. 1a^1^. Using mirror-based reflective coverslips, images of four complementary views were obtained in 250 ms simultaneously, and the imaging efficiency and speed were boosted by a factor of 2. Notably, mirror-enhanced dual-view LSFM improved the spatiotemporal resolution and collection efficiency with no additional hardware requirements and reduced the number of objectives from 4 to 2^2,3^. With a modified deconvolution algorithm, a spatial resolution as high as 300 nm in the *x*, *y*, and z dimensions was achieved. Mirror-enhanced dual-view LSFM enables high speed, high-resolution imaging of biological specimens.

**Fig. 1:**
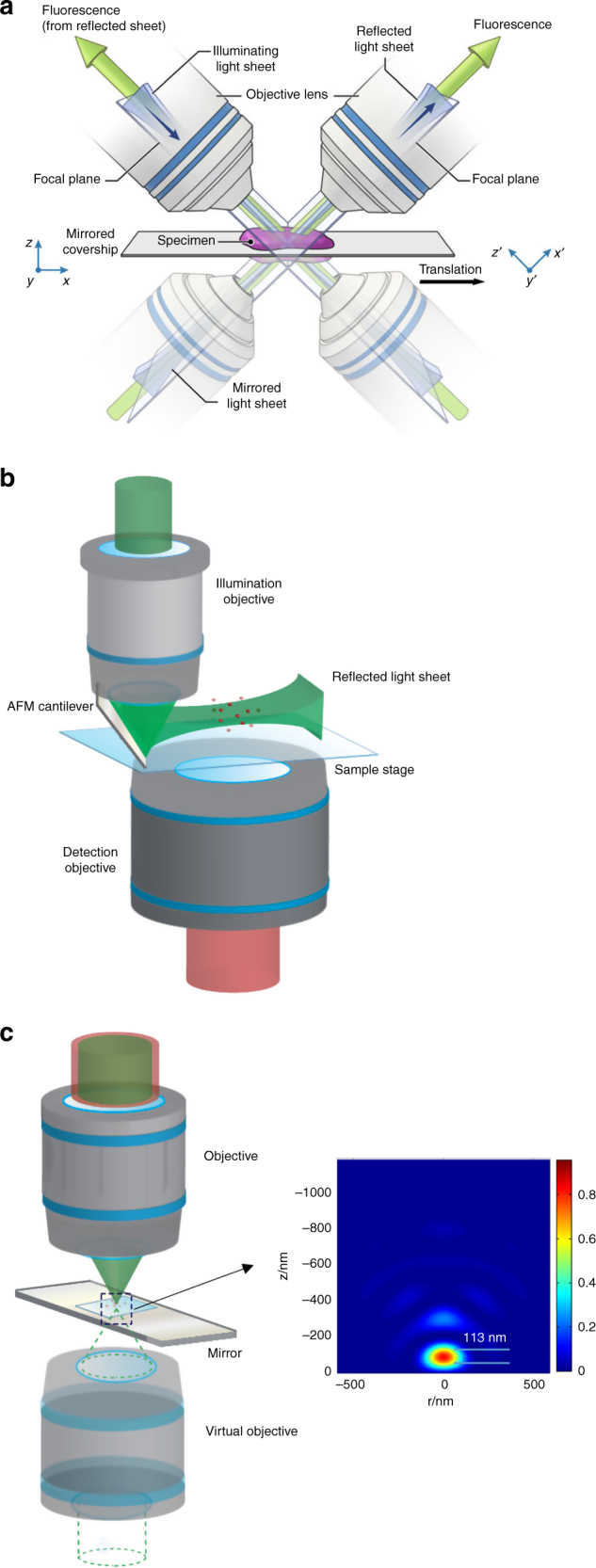
Mirror-enhanced optical microscopy. **a** Mirror-enhanced dual-view light sheet microscopy duplicates the conventional iSPIM to tetrad objectives. **b** Reflected light sheet microscopy folds the upper objective’s excitation to form a thin light sheet. **c** Mirror-enhanced super-resolution microscopy generates an interferometric focal spot. **a** and **c** inset are adapted from refs.^1, 6^

Mirrors not only can reduce the number of objectives in light sheet microscopy but also can fold optical path to reduce the size. Previously, the resolution of light sheet microscopy was limited by the size of the objectives because they should be placed perpendicularly and as close as possible to each other. This restriction can be solved by changing the “real” objective to an “imaginary” objective by using mirror reflection. In reflected light sheet microscopy (RLSM) developed by Sunney Xie’s group at Harvard University^4^, a mirror is used to bend the light path such that the illumination and detection objectives are not orthogonal, as in other light sheet microscopes, but are opposite to each other. In this case, the physical size of the objective is no longer a barrier for the application of high-numerical aperture objectives. RLSM possesses the advantages of superior SBR, fast image acquisition speed, low phototoxicity, and optical sectioning capability. A thin light sheet of 0.5 μm can be created, with fivefold better SBR, thereby enabling resolution of DNA-binding dynamics with a temporal resolution of 100 Hz. In addition, combining the blinking photophysics of rhodamine-based dyes, RLS illumination microscopy can be upgraded to reflective light sheet illumination super-resolution microscopy^5^.

Mirrors can benefit microscopy not only in geometrical optics but also in wave optics. For example, a mirror can cause interference and standing waves, similar to those in a laser cavity. Peng Xi’s group at Peking University generated an axial narrowed focal spot in laser scanning microscopy by adding a mirror beneath the specimen^6,7^. In this technique, termed MEANS, a confocal microscope can be converted into an effective 4Pi microscope without additional cost. The interference greatly enhanced the local intensity, which makes MEANS-STED capable of resolving the porous structure of the nuclear pore complex. Moreover, in such a configuration, axial information about the cellular organelle can be obtained through the fluorescence lifetime, which is modulated by the surface plasma resonance of the mirror^8^.

In the future, mirror-enhanced dual-view LSFM and RLSM can be combined with single-molecule localization microscopy, super-resolution optical fluctuation imaging, and Bayesian analysis localization microscopy to achieve three-dimensional nanoscopic dynamics imaging in living cells. The MEANS approach has potential applications in fluorescence correlation spectroscopy, fluorescence lifetime imaging microscopy, fluorescence recovery after photobleaching, and pump-probe-based microscopy technologies such as coherent anti-Stokes Raman scattering, stimulated Raman scattering, and transient absorption microscopy^9^.

As a fundamental optical element, mirrors can greatly enhance the performance of optical microscopy. The unique view and resolution can help biological scientists observe live cell structures at improved resolution in both the spatial and temporal dimensions. The improvements offered by using mirrors for microscopy can smoothly accelerate fluorescence imaging applications in the realm of live cell dynamics studies.

## References

[1] Wu YC (2017). Reflective imaging improves spatiotemporal resolution and collection efficiency in light sheet microscopy. Nat. Commun..

[2] Wu YC (2013). Spatially isotropic four-dimensional imaging with dual-view plane illumination microscopy. Nat. Biotechnol..

[3] Kumar A (2014). Dual-view plane illumination microscopy for rapid and spatially isotropic imaging. Nat. Protoc..

[4] Gebhardt JCM (2013). Single-molecule imaging of transcription factor binding to DNA in live mammalian cells. Nat. Methods.

[5] Zhao ZW (2014). Spatial organization of RNA polymerase II inside a mammalian cell nucleus revealed by reflected light-sheet superresolution microscopy. Proc. Natl Acad. Sci. USA.

[6] Yang XS (2016). Mirror-enhanced super-resolution microscopy. Light Sci. Appl..

[7] Graydon O (2016). Microscopy: axial super-resolution. Nat. Photonics.

[8] Chizhik AI, Rother J, Gregor I, Janshoff A, Enderlein J (2014). Metal-induced energy transfer for live cell nanoscopy. Nat. Photonics.

[9] Wang P (2013). Far-field imaging of non-fluorescent species with subdiffraction resolution. Nat. Photonics.

